# The search for novel insecticide targets in the post-genomics era, with a specific focus on G-protein coupled receptors

**DOI:** 10.1590/0074-02760160345

**Published:** 2017-01

**Authors:** Michelle Ngai, Mary Ann McDowell

**Affiliations:** University of Notre Dame, Eck Institute for Global Health, Department of Biological Sciences, Notre Dame, USA

**Keywords:** genomics, insecticides, G-protein-coupled receptors

## Abstract

Insects are considered pests globally, implicated in the destruction of agricultural fields and transmission of pathogens that cause deadly human diseases, such as dengue, Zika and malaria. The diversity of the insecticide arsenal has remained stagnant for decades, but the recent rise of insecticide resistance fueled the discovery of novel modes of action, and the power of genomics has reinvigorated this search. This review discusses the importance of comparative and functional insect genomics in the identification of potential gene targets for an insecticidal mode of action with low off-target toxicity. Due to the global participation in the sequencing and annotation of insect genomes, the targeting of specific genes with molecular tools like RNAi and CRISPR/Cas9 for genome engineering and consequent functional identification and validation has become more efficient. While there are multiple avenues to explore for insecticidal candidates, this review identifies G-protein coupled receptors as attractive targets, and hones in on the octopamine and dopamine receptors due to their potential.

Insects are vital to the overall order of many ecosystems and have lived collectively with the human population for centuries. While insects are viewed as beneficial in several facets of society, they also endanger the human population. As agricultural pests, insect species have been the cause of food source damage and depletion, resulting in substantial economic losses. Moreover, insects serve as reservoirs for several pathogens, the causative agents of many debilitating human diseases. Dengue fever, malaria, and leishmaniasis are a few examples of human diseases transmitted by insect vectors, afflicting high morbidity and mortality on the global population, and disproportionately burdening the less-developed regions of the world due to optimal climate conditions for vector survival and reproduction, infrastructure conditions and the lack of sustained control programs. Therefore, considerable efforts, on both global and local levels, have been placed on vector control with an integrative strategy: implementation of preventive measures through community engagement including the elimination of breeding grounds and the use of personal protective gear, complemented with the dissemination of insecticides, and/or alternative approaches such as genetic manipulation. Promising control results have been short-lived, however, with the development of insecticide resistance, the culmination of factors including pesticide misuse, lack of novel compounds in the pipeline, and a dearth of diversity in the mode of action. Insecticide resistance has been reported in areas worldwide, with the most commonly used compounds such as synthetic pyrethroids, organophosphates, and chlorinated hydrocarbons, being less effective in targeting and altering the insect nervous system (WHO 2014). Despite this rise in resistance over the years, the response for advancing the novel insecticide pipeline has been weak, and consequently, few new compounds have been released for public health purposes in the last 30 years. Insecticide resistance, therefore, has been a contributing factor to continued persistence of agricultural pests and the resurgence of vector-borne diseases, highlighting the importance of (i) identifying and developing insecticides with alternative modes of action and (ii) alternative control approaches.

While the scientific development of new insecticides has plateaued, great strides have been made in the field of insect genomics. Technology advancements and lower costs have enabled more efficient and accurate sequencing of whole genomes, piquing interest in genome projects and the utilisation of resultant data to answer biological questions related to evolution, physiology, development, and immunity. The large-scale sequencing of vector genomes has intensified in recent years, following the completion of the *Anopheles gambiae* genome. Recognising the critical implications of such work, the National Institute of Allergy and Infectious Diseases and National Institutes of Health established a Bioinformatics Research Center (BRC) to maintain an open-access database of sequenced genomes along with structural and functional annotations. VectorBase, a branch of the BRC, was funded in 2004 to specifically focus on invertebrates responsible for the transmission of human disease. Currently, VectorBase accommodates the data for 60 organisms including 28 mosquito species of the genera *Aedes*, *Anopheles* and *Culex*, two sand fly species (*Lutzomyia longipalpis* and *Phlebotomus papatasi*), six tsetse fly species (*Glossina morsitans*, *G. austeni*, *G. brevipalpis*, *G. fuscipes*, *G. pallipdipes*, and *G. palpalis*), and the kissing bug (*Rhodnius prolixus*). More recently, the i5K initiative was launched with the mission of sequencing the genomes of 5000 arthropod species over five years. “High-priority” candidates were identified based on several factors including their impact on the human population via health, food supply and economic security. Complete and draft genomes, in addition to ongoing work are available at i5K workspace@NAL. International consortiums have assembled a multitude of additional databases, notably Flybase, Hymenoptera Genome Database, BeetleBase, and most recently, InsectBase. These databases are invaluable resources, providing entomologists with a vast array of genomic data, both structural and functional, and thus assists with comparative studies on global genome architecture and evolution, in addition to the identification of genes associated with host preference, insecticide resistance, or immunity. The advent of the post-genomics era, therefore, has been contributing towards the improvement of global health by (i) aiding in the identification of new targets for novel insecticides and vaccines in the combat against vector-borne diseases, and (ii) improving the understanding of specific genes and mechanisms underlying pathogenicity of microbes and insecticide resistance.

Data mining and bioinformatics tools have become critical in the analysis and interpretation of the biological datasets attained from large-scale sequencing efforts. These tools have been widely utilised for targeted therapeutics in the human population, more specifically to identify (i) the genetic variation between populations and (ii) biomarkers for pathogenesis and progression of particular disease (Mansmann 2005). While the myriad of advantages to this approach is recognised across applications, with insecticide development benefiting in a similar manner to human drug development, there is limited exploitation. Insect genomics, however, has enabled the discovery of insecticide targets via functional characterisation of proteins that are essential to survival or other biological functions. More importantly, the power of genome sequences and comparative genomics allows the capitalisation of slight differences between species, potentially driving insecticide development towards “designer insecticides” with high specificity for an individual insect and low toxicity for non-target species.

The introduction of draft insect genomes with the respective gene annotations has played a crucial role in the mining of protein targets for insecticides. Conventional neuroactive insecticides have targeted acetylcholinesterase, nicotinic acetylcholine receptors and voltage-gated ion channels, but G-protein coupled receptors (GPCRs), kinases, ATPases, synthases and carboxylesterases have also demonstrated potential as novel insecticide targets. Comparative genomics has aided in the detection of gene targets through homologous sequences, and as a model organism, knowledge surrounding *Drosophila melanogaster* is the most robust, providing a good source of comparison for gene prediction and annotation. Due to its notoriety as an agricultural pest, Wang et al. (2014) sequenced the complete genome of *Locusta migratoria* with paired-end sequencing libraries on the Illumina platform. *De novo* genome assembly resulted in a 6.52 Gb genome while gene model predictions based on four insect reference gene sets (*D. melanogaster*, *Apis mellifera*, *Acyrthosiphon pisum*, and *Pediculus humanus*) indicated the existence of 17,307 genes. A total of 290 genes were described as potential insecticide gene targets; 124 were noted as either a GPCR or ion channel, while the other 166, including synthases, carboxylesterases, ATPases and kinases, were identified after *in silico* screening for orthologues of lethal genes in *Caenorhabditis elegans* and *D. melanogaster* (Wang et al. 2014). Similarly, Karatolos et al. (2011) generated the transcriptome for two strains of the whitefly *Trialeurodes vaporariorum* through 454 pyrosequencing. After classifying the function of the putative proteins, transcripts encoding traditional insecticide targets, including the acetylcholinesterase enzyme, nicotinic acetylcholine receptor subunits, acetyl-CoA carboxylase, voltage-gated sodium channel, g-aminobutyric acid receptor, glutamate-gated chloride channel, and ryanodine receptor, were identified (Karatolos et al. 2011). Additionally, Nene shed light on the genome of *Aedes aegypti* by whole-genome shotgun sequencing purified larval DNA. This played a key role in the functional classification of putative proteins through computational analysis of domains, secretion signaling sequence and transmembrane motifs, and more significantly, identification of transcripts encoding 135 GPCRS that are potential insecticide targets (Nene et al. 2007).

Comparative genomics has also aided in the elucidation of the molecular mechanisms underlying insecticide resistance, including metabolic resistance and target-site resistance. Metabolic resistance is characterised by alterations in expression and activity of detoxification enzymes involved in the insecticide biodegradation process, including carboxyl/choline esterases (CCE), glutathione-S-transferases (GST), cytochrome P450 monooxygenases (P450), and ATP-binding cassette (ABC) transporters (Hemingway et al. 2004). Due to their involvement in insecticide resistance, these proteins have been identified in many insect genomes. Based on BLAST queries and manual annotation with respect to *D. melanogaster*, a total of 173 and 235 putative detoxification genes were identified in the genomes of human disease vectors, *An. gambiae* and *Ae. aegypti*, respectively. The *An. gambiae, Ae. aegypti*, and *D. melanogaster* species have 106, 161, and 85 P450 transcripts identified, respectively (Ranson et al. 2002, Strode et al. 2008). The crop pests, *T. vaporariorum* and *Ac. pisum,* also have a number of enzymes for insecticide metabolism. Fifty-seven transcripts encoding P450, 27 CCEs and 17 GSTs were detected in the *T. vaporariorum* transcriptome, and correspondingly, 83 putative P450, 29 CCE and 20 GST proteins were discovered in the pea aphid, *Ac. pisum* (Ramsey et al. 2010, Karatolos et al. 2011)*.* The ABC transporter family has also been annotated in several arthropod genomes, including 73, 52, 49, and 41 genes being detected in *T. castaneum*, *An. gambiae*, *Ae. aegypti*, and *A. mellifera* (Dermauw & Van Leeuwen 2014).

The analysis of copy number variation and identification of polymorphisms has aided in the discovery of gene amplification and overtranscription of detoxification genes in resistant insect populations. In highly insecticide-resistant *Culex pipiens quinquefasciatus*, the copy number of carboxylesterase enzymes *esta2* and *estb2* is increased by up to 80 fold. Elevated level and activity of cytochrome P450 is often found in insecticide-resistant mosquitoes as a result of constitutive overexpression regulated by cis- and trans-acting factors. The upregulation of genes within the *Cyp6* P450 family, more specifically, has been implicated in the insecticide-resistant mosquitoes. Elevated transcripts of these genes have been observed in pyrethroid-resistant strains of *An. gambiae* in East Africa and permethrin-resistant *Culex* mosquitoes (Hemingway et al. 2004). Similar gene amplification and upregulation in transcription activity is observed for GST enzymes; a specific GST involved in dehydrochlorination has been detected to function at a higher rate in DDT-resistant *Ae. aegypti* and *An. gambiae* mosquitoes (Grant et al. 1991, Prapanthadara & Ketterman 1993). The *Meligethes aeneus* transcriptome, the European pollen beetle responsible for the decline of rapeseed crop, illustrates a comparable pattern. After mapping RNAseq reads and conducting a GO analysis of susceptible and pyrethroid-resistant beetle populations, Zimmer et al. (2014) identified three differentially expressed GST genes in all resistant populations (Zimmer et al. 2014).

Target-site resistance, on the other hand, is characterised by polymorphisms within the target region, functionally changing the protein and its ability to induce a mode of action. Typically, organophosphates and methylcarbamate insecticides target the serine residue within the active site gorge of acetylcholinesterase, preventing acetylcholine hydrolysis (Hemingway et al. 2004). However, through comparative sequencing of susceptible and resistant mosquitoes, specific amino acid substitutions within the coding region of the AChE1 catalytic site have been described in insecticide-resistant mosquitoes. The mutations are found to constrict the opening of the active site, restricting access to the catalytic residues. More specifically, a glycine to serine substitution has been a consistent find in several mosquito species, including *An. gambiae* and *C. quinquefasciatus*, causing the enzyme to be less sensitive or completely insensitive to the insecticides (Weill et al. 2003). The current acetylcholinesterase target is ubiquitous in all animals, causing high off-target toxicity. However, the availability of AChE sequences for multiple species including insects, birds, amphibians and mammals has enabled the identification of a potential target specific to insects. Sequence alignments of 134 different species have revealed a cysteine residue conserved in 22 insects, primarily those that transmit human diseases such as mosquitoes *Ae. aegypti*, *An. gambiae*, *C. pipiens*, and the sand fly, *Lu. longipalpis,* and those considered to be crop or residential pests including *Tribolium castaneum*, *Bombyx mori*, and *Aphis gossypii* (Pang et al. 2012). Pyrethroids, a common insecticide class, act by binding to voltage-gated sodium channels and altering its gating kinetics. Functional changes in the sodium channel have led to the development of knockdown resistance (*kdr*) in multiple insect species*.* Single and double nucleotide substitutions have been reported to modify the structure, either diminishing or altogether eliminating the insecticide’s binding affinity to the target proteins. First described in the model system of the house fly, *Musca domestica L*, the molecular mechanism underlying *kdr* has been well-investigated in disease vectors and agricultural pests. Analysis of sequence alignments of susceptible and *kdr* houseflies highlighted two specific missense point mutations, including a leucine to phenylalanine at residue 1014 of the sodium channel gene. Other species were examined for a similar gene mutation; the common *kdr* mutation has been identified in seven species, including West African pyrethroid-resistant *An. gambiae* and *C. pipiens*. Characterisation of the sodium channel has led to the identification of over 20 unique polymorphisms of pyrethroid-resistant species. The leucine is substituted with a serine in the east African disease vector populations of *An. gambiae*, while other discoveries include a L925I, L1014H and T929I in *B. tabaci*, *H. virescens* and *P. xylostella* resistant phenotypes, respectively (Soderlund & Knipple 2003).

Recently, investigations for new insecticide modes of action have surged, and RNA interference (RNAi) has facilitated in narrowing the search to specific protein targets by aiding in the functional and behavioural characterisation of genes. RNAi mediated gene silencing is often completed via microinjection of double-stranded RNA or small interfering RNA, but uptake through topical application and ingestion have also demonstrated to be worthwhile. One group, in particular, optimised the methodology for mosquito larval ingestion by incorporating chitosan/interfering RNA nanoparticles into the typical fish food and dry yeast mixture. After targeting the *Ae. aegypti semaphorin-1a* gene with these nanoparticles, quantitative reverse-transcriptase polymerase chain reaction (qRT-PCR) was performed and a reduced level of expression, approximately 60%, was detected in larval brains. Similar results were observed when the nanoparticles were used to target the transcription factor, singled-minded (Zhang et al. 2015). The RNAi technique is often utilised to identify targets that are essential to function; by silencing the target’s expression, there is potential to disrupt growth and development, causing early mortality. A RNAi screen was conducted for *T. castaneum* to prioritise RNAi target genes in insects by Ulrich and colleagues. The iBeetle database was mined, and dsRNA described to elicit mortality post-injection was selected for further investigation. A titration curve of selected dsRNA was conducted to identify and validate the optimal response. Furthermore, similar experiments were implemented for the orthologues of RNAi target genes reported to cause high mortality in the western corn rootworm. Overall, the group identified 11 novel targets which displayed fatality. Similarly, Allenza and colleagues focused on the cotton aphid’s matrix metalloproteases family for RNAi investigation due to its reported involvement in embryonic lethality. By targeting these genes with RNAi machinery, paralysis was induced in the larval and pupal stages, demonstrating its potential as a novel insecticide target (Allenza & Eldridge 2007). While premature mortality via RNAi targeting is a desired result, the molecular tool can be applied to disrupt other key functions. For example, the *Ae. aegypti*, *An. gambiae*, and *C. pipiens quinquefasciatus* genomes were analysed for orthologues of known CO_2_ receptors in *D. melanogaster*. Two specific *Ae. aegypti* CO_2_ receptor genes were functionally validated via RNAi experiments; a reduction in the expression of the genes curtailed the mosquitoes’ ability to respond to CO_2_ (Erdelyan et al. 2012). Due to the important role of CO_2_ sensing in the blood-feeding female mosquitoes’ ability to seek a host, diminishing or outright abolishing this capacity has the potential to reduce disease transmission, proving to be an avenue worth further exploration.

While RNAi has demonstrated its utility in identification of control targets, this technique has also been recently tested as an innovative biological control strategy. In lieu of genetically modifying mosquitoes with the sterile insect technique, Whyard established an alternate method for mosquito sterilisation and sex sorting via RNAi. Ingestion of dsRNAs targeting testis and female sex determination genes by *Ae. aegypti* larvae resulted in mature males with reduced fertility but strong ability to compete in mating with the wildtype, and a male-biased population (Whyard et al. 2015). This RNAi methodology has also been effective in the agricultural realm. Baum identified 14 genes within *Diabrotica virgifera* that are essential for its survival, and developed a transgenic corn expressing dsRNA targeting one specific gene, the midgut enzyme vacuolar ATPase. Results indicate that the dsRNA expression has a protective effect on the corn; a reduced infestation of the agricultural pest is the outcome of the transgenic corn line. Similarly, Mao fed *H. armigera* larvae plant material expressing dsRNA specific to a P450 gene. Since this gene appears to play a role in resistance to gossypol, a natural insecticide produced by the cotton plant, gene silencing in *H. armigera* consequently led to increased sensitivity to the compound and delayed larval growth (Gordon & Waterhouse 2007). While this RNAi strategy has not been introduced into field practice as a form of biological control, these experimental results demonstrate its potential.

CRISPR/Cas9 is another influential biological tool utilised for genome editing and engineering. Since its introduction in mammalian cells, this system has been widely adopted and applied to many model and non-model organisms, including several insects, notably *D. melanogaster*, *Ae. aegypti*, *An. gambiae*, *B. mori*, and *T. castaneum*. Guide RNA and the cas9 nuclease are delivered to induce double-stranded breaks at targeted sequence regions within the genome, perturbing the translation process and consequent protein. This relatively new methodology, therefore, facilitates the study of gene function both in terms of identification and validation, and can inform on suitable gene targets for novel insecticides. For example, targeting the *Nix* gene of *Ae. aegypti* with the CRISPR/Cas9 combination demonstrated it is fundamental to male development (Hall et al. 2015). Genes responsible for insecticide resistance were further validated in *C. quinquefasciatus*; mutations within the cytochrome P450 CYP9M10 gene produced more pyrethroid-sensitive individuals, confirming its role in pyrethroid resistance (Itokawa et al. 2016). Finally, the knockout of gene *abdominal-a* in *Plutella xylostella* led to severe abdominal deformities and significant lethal ramifications, exposing its potential as a mode-of-action for insecticide targeting (Huang et al. 2016).

While there are a host of unique mode of actions currently being scrutinised, GPCRs are an attractive target. This protein family is widely expressed in mammals and modulates a wide spectrum of biological processes including development, visual, gustatory, and olfactory sensing, homeostasis, and hormonal regulation. In invertebrates, specifically, GPCRs have been found to regulate physiological pathways relevant to development, reproduction, metabolism, and ecdysis. As mediators of such crucial physiological mechanisms, GPCRs are considered to be lucrative drug targets. Recently, many groups have started exploiting GPCRs in the development of novel insecticides and studies indicate there is high potential. By modifying or disrupting GPCRs within the host organism, core cellular functions and/or responses are either blocked or over-stimulated resulting in reduced fitness or reproductive capacity, and potentially death.

Ubiquitous in almost all eukaryotic organisms, GPCRs are considered one of the largest families of membrane-spanning proteins. All GPCRs share a common architecture consisting of a seven-transmembrane (TM) a-helical core linked to six alternating intracellular and extracellular loops with the amino and carboxy terminals lying on the extracellular and cytoplasmic sides, respectively. Both conserved and divergent residues are observed, with conserved amino acids within the transmembrane domains and variable regions within the loops. For example, a conserved arginine residue produces a D-R-Y motif at the cytoplasmic side of transmembrane three throughout all members of the rhodopsin-class (Gether 2000). Despite a few highly conserved residues, the overall homology is low, allowing differences to be exploited in the prevention of off-target effects, but also limiting the ability of similarity-based searches in the mining of GPCRs within insect genomes. Therefore, groups moved towards the exploitation of distinctive topological characteristics in the development of valuable bioinformatic predictive tools. Computational algorithms such as GPCRHMM, Phobius and Quasi-periodic Feature Classifier have been trained to scan individual amino acid sequences for conserved topology features i.e. seven hydrophobic transmembrane regions, a distinct amino acid length, as well as the composition and length of cytosolic and extracellular loops (Wistrand et al. 2006). An innovative classifier, Ensemble* was designed recently to detect GPCRs specifically in insect vectors by merging the predictive capabilities of algorithms, GPCRHMM and Pfam A GPCR clan Hidden Markov Models, and utilising a combined overall likelihood score. Primed with GPCR sequences in *Ae. aegypti, An. gambiae*, *A. mellifera*, *D. melanogaster*, and *P. humanus*, in addition to, well-characterised *Homo sapiens,* the Ensemble* classifier proved particularly adept for vector species by identifying 52 GPCRs that were not previously known (Nowling et al. 2013). These tools have led to the discovery of over 200 GPCRs, many of which have high sequence similarity to known drug targets, and provide a strong repertoire for the insecticide development pipeline.

As proven by multiple insect genome annotations, GPCRs are abundant and available for insecticide development investigation (Table); the research, however, remains limited and severely under-resourced. The biogenic amine GPCR family including octopamine, tyramine, dopamine, and serotonin receptors, however, has recently gained interest due to its pervasive presence in invertebrates. With full-length sequences readily accessible from the genome databases, de-orphanisation and functional/pharmacological characterisation has been achieved for a multitude of GPCRs.

**TABLE:** Potential G-protein coupled receptor targets for novel insecticide modes of action. Outlined are the putative biogenic amine and neuropeptide receptor genes for *Aedes aegypti*, *Anopheles gambiae*, *Culex quinquefasciatus*, *Glossina morsitans*, and *Ixodes scapularis*

Species	Gene accession number			

	Octopamine /tyramine receptors	Dopamine receptors	Serotonin receptors	Neuropeptide receptors
*Ae. aegypti*	AAEL016990	AAEL003920	AAEL011844	AAEL010626
	AAEL004396	AAEL005834	AAEL009573	AAEL007924
	AAEL004398	AAEL005945	AAEL017162	AAEL013505
	AAEL014224	AAEL014373	AAEL017126	AAEL015418
	AAEL006844	AAEL017166	AAEL008360	AAEL008296
	-	AAEL005952	AAEL017019	AAEL017005
	-	-	AAEL017272	AAEL012190
	-	-	AAEL002717	-
	-	-	AAEL015553	-
*An. gambiae*	AGAP012698	AGAP004613	AGAP004222	AGAP004122
	AGAP002519	AGAP000667	AGAP002232	AGAP004123
	AGAP000045	AGAP004453	AGAP002229	AGAP012378
	AGAP000606	AGAP002888	AGAP007136	AGAP007924
	-	-	AGAP011481	AGAP000351
	-	-	AGAP004223	AGAP002881
	-	-	-	AGAP000115
*C. quinquefasciatus*	CPIJ019015	CPIJ000649	CPIJ015747	CPIJ014103
	CPIJ013218	CPIJ013660	CPIJ011883	CPIJ016696
	CPIJ013660	CPIJ015294	CPIJ011881	CPIJ018504
	CPIJ019013	CPIJ000651	CPIJ003684	CPIJ016508
	CPIJ007716	-	CPIJ013433	CPIJ013069
	CPIJ007715	-	CPIJ019015	CPIJ011717
	CPIJ007717	-	CPIJ011755	CPIJ006984
	-	-	-	CPIJ018265
*G. morsitans*	GMOY009784	GMOY002017	GMOY004778	GMOY006767
	GMOY002392	GMOY003245	GMOY007454	GMOY006636
	GMOY003496	GMOY004240	GMOY011398	GMOY011997
	GMOY002031	-	GMOY012096	GMOY012305
	GMOY005183	-	GMOY001739	-
	GMOY006177	-	GMOY012148	-
	-	-	GMOY011995	-
*I. scapularis*	ISCW003835	ISCW001496	ISCW019072	ISCW000923
	ISCW005195	ISCW008775	ISCW019070	ISCW000924
	ISCW013655	ISCW005105	ISCW017050	ISCW020603
	ISCW008522	ISCW006077	ISCW020906	ISCW003493
	ISCW004650	ISCW008755	ISCW002246	-
	ISCW000606	ISCW008917	ISCW006710	-
	ISCW007598	ISCW015254	ISCW007619	-
	ISCW008201	ISCW008917	-	-
	ISCW009595	-	-	-
	ISCW013545	-	-	-
	ISCW014824	-	-	-
	ISCW021342	-	-	-

Highly prevalent in invertebrates and found only in trace amounts within mammals, octopamine signaling is of particular importance for insecticide targeting considering that off-target effects and vertebrate toxicity can be avoided. Octopamine functions as a neurotransmitter and neuromodulator in the central nervous system and as a neurohormone when released into the hemolymph system. By binding to its respective GPCR, a signaling cascade is initiated, influencing essential physiological processes (*i.e.* metabolism, phase transition, immune response) and behavioural states (*i.e.* feeding, pheromone response) (Gross et al. 2014). Currently, there is one insecticide recognised by the Insecticide Resistance Action Committee that acts upon the octopamine receptor in a broad spectrum of invertebrates. By mimicking the native ligand, Amitraz acts as an agonist to the receptor and over-stimulates neuronal responses, resulting in paralysis and death (Robb et al. 1994). While this chemistry has been effective towards certain insect vectors and agricultural insect pests, efforts of other groups are paving the way forward for the identification of other chemistries that can act in a similar manner. A previous study utilised a combination of in vitro and *in silico* methodologies to identify a specific scaffold that carries strong potential for insecticidal activity via the octopamine receptor in *An. gambiae*. First, a list of chemistries and their effects on the octopamine receptor were identified, and subsequently a computational model was developed and trained to screen approximately 12 million compounds in the ZINC online database by looking at its binding activity. Promising insecticidal candidates were selected, and further validated in vitro. Additionally, an aspartic acid in TM3 was identified to be vital to proper ligand binding and receptor functionality; site-directed mutagenesis replaced the aspartic acid with an alanine or asparagine, completely suppressing activity (Kastner et al. 2014). A number of other studies provide the pharmacological profile of the octopamine receptor in insect species including, but not limited to *B. mori*, *L. migratoria* and *D. melanogaster* (Verlinden et al. 2010).

Dopamine receptors have also been the subject of comprehensive study for insecticide discovery. Dopamine functions in a similar manner to octopamine, triggering a signaling pathway and a host of physiological mechanisms. Results from studies conducted in *D. melanogaster*, *T. castaneum* and *Ae. aegypti* demonstrate the dopamine receptors’ role in larval growth, neuronal development and other important life stages. RNAi knockdown of the receptors not only prevented development, but inhibited larvae from undergoing ecdysis, leading to high rates of mortality in the premature stages (Regna et al. 2016). High levels of dopamine immediately following certain life events also reveals involvement in other processes; dopamine spikes post bloodmeal and in newly-moulted adults suggesting involvement in ovarian and/or egg development and also in the sclerotisation process, respectively (Andersen et al. 2006). Finally, expression levels of the receptor have been discovered in the salivary gland, indicating a role in salivation, an important process for host-seeking, feeding, duration of probing, immunity, and survival (Regna et al. 2016). Unlike octopamine receptors, dopamine receptors are present in both invertebrates and mammals. However, this should not undermine their potential as insecticide targets. A group has focused on pharmacologically characterising the dopamine receptors in *Ae. aegypti*. After conducting a screen of the LOPAC1280 library, 51 compounds with potent antagonistic activity and the potential to disrupt homeostasis were identified. A follow-up study scrutinising the effect of the compounds in vivo exposed the toxicity of four specific scaffolds from tricyclic antidepressant and antipsychotic classes; lethality was evident in both immature and mature stages of *Ae. aegypti*. To negate the possibility of cross-reactivity of these chemistries on the human population, the selectivity of eight compounds for the *Ae. aegypti* dopamine receptor versus the human orthologue was analysed. Based on in vitro experiments, certain compounds were significantly more potent in the vector, suggesting their feasibility and suitability for the insecticide development pipeline. More importantly, these findings support the concept of “designer insecticides”, with small sequence differences between species capable of altering compound binding affinity (Conley et al. 2015).

While octopamine and dopamine receptors are examples of novel modes of action due to their involvement in critical biological processes, there are numerous of other suitable target candidates within the GPCR superfamily. Two specific examples include the serotonin (5-HT) receptors and neuropeptide receptors. Expression patterns of the 5-HT receptor and its ligand indicate a role in the modulation of a multitude of features including salivation, feeding behaviour, locomotion, learning and memory, and circadian rhythms (Vleugels et al. 2015). On the other hand, neuropeptide receptors include a suite of families involved in the regulation of the moulting process, growth, reproduction, energy metabolism, and feeding behaviour (Van Hiel et al. 2010). With roles imperative to biological function, these two receptor families are additional examples of targets requiring further attention and experimentation. Overall, though this review focuses on GPCRs, similar tools and methodologies can be adapted and applied to other potential targets outside of this superfamily, such as voltage-gated ion channels, receptor tyrosine kinases and guanylyl cyclases.

The global efforts towards genome sequencing are unprecedented, and the benefits are recognised in multiple areas of the scientific community. This post-genomics era, however, has greatly accelerated the search for novel insecticide targets. Easily accessible, complete gene sequences allow for the pharmacological and functional characterisation of putative targets, and ultimately, the identification of promising candidates for the insecticide development pipeline. Subtle sequence differences between species have also been detected and can be exploited for the advancement of “designer insecticides”. Insects have been challenging the human population through food depletion and disease transmission for centuries, but the power of new and improved genomics is supporting our ability to identify and evaluate numerous targets for development into safe and sustainable solutions.
